# A long, winding trajectory of suffering with no definite start and uncertain future prospects – narratives of individuals recently diagnosed with fibromyalgia

**DOI:** 10.1080/17482631.2022.2056956

**Published:** 2022-03-31

**Authors:** Anne Marit Mengshoel

**Affiliations:** Department of Interdisciplinary Health Sciences, Institute of Health and Society, Medical Faculty, University of Oslo, Oslo Norway

**Keywords:** Fibromyalgia, patient experiences, illness, suffering, narrative methods

## Abstract

**Purpose:**

Fibromyalgia is a contested illness with unknown aetiology and poorly understood development. The present aim is to explore the pre-diagnostic illness trajectory narrated by individuals recently diagnosed with fibromyalgia (FM).

**Methods:**

Individual interviews about the course of the illness were conducted with seven women and three men (age from early 20s to 50s) who had recently been diagnosed with FM. A narrative analysis of what the interviewees told and how the stories were narrated was conducted.

**Results:**

The findings are expressed by three storylines. “Strenuous life and alerted body preluding illness” displays a difficult, unsupported life and bodily sensitivity to stimuli. “Recurrent pains unfolding to become a lasting and complex illness” describes individuals pushing themselves to meet social obligations until they come to a full stop. “Diagnosed but still uncertain presence and future” portrays satisfaction with finally being diagnosed with FM and being supported by others, but still there are no solutions as to do about it.

**Conclusions:**

The three storylines portray a long, winding trajectory of suffering starting before the onset of illness, and unfolding illness gradually becoming persistent and overwhelming. Finally, a diagnosis of FM is arrived at, but how the situation will evolve is uncertain.

## Introduction

FM is a common disorder (Heidari et al., 2017) characterized by widely distributed persistent bodily pain, excessive fatigue and an array of other complaints and problems, such as unrefreshed sleep, concentration and memory problems, depression, headache, irritable bowel, and impaired daily functioning (Arnold et al., 2008). Several biological interacting mechanisms, genetic vulnerability and psychological changes are suggested to explain the symptoms (Arnold et al., 2019; Littlejohn & Guymer, 2020). Nevertheless, there are controversies about the nature, existence, diagnosis and treatment of the FM condition (Barker, 2011; Bidari et al., 2018). FM cannot be verified clinically by any laboratory or radiological measures (Jutel, 2011), and no known cure or lasting effective treatments are available (Macfarlane et al., 2017). Thus, it is debated whether diagnosing people with FM leads to medicalization of trivial health complaints (Hadler, 1996), and the diagnosis has low prestige within the field of medicine (Album & Westin, 2008). Nevertheless, patients with FM are frequent users of health services (Mengshoel et al., 2022), but as indicated by the title of the paper by Doebl et al. (2020), “no one wants to look after the fibro patient”. One plausible reason is that FM is difficult both to understand and treat.

The symptoms of FM mimic those of several other diseases. Accordingly, the diagnostic process often includes years of consultations with several medical specialists to exclude other diseases (Choy et al., 2010; Perrot et al., 2012). A meta-synthesis of qualitative studies portrays the diagnostic journey as distressing and shifting from hope for an explanation and medical help to hopelessness when negative results occur (Mengshoel et al., 2018). When several other diseases are excluded, questions about whether the symptoms are imagined or only an exaggeration of trivial complaints can be raised by both health professionals and others, and thereby, patients’ moral agency is put at stake (Mengshoel et al., 2018). According to the responses of 941 British patients with FM, the patients themselves relate their debut of FM to physical assaults (e.g., injuries, surgery, side-effects of drugs), emotional trauma and distress (e.g., loss, abuse, bulling, depression), or stress and vulnerability (e.g., burden of care, high work demands, personal stubbornness, not giving up, heredity, growing pains) (Furness et al., 2018). This suggests that patients relate the reason for getting FM to events occurring in their life, but how the patients experience the development of illness before ending up with a diagnosis of FM is still not illuminated.

Several qualitative studies have examined what it is like to be ill with FM. A meta-synthesis describes the illness experiences of patients diagnosed with FM under the following themes (Sim & Madden, 2008): the experience of symptoms, legitimacy, and coping. This meta-synthesis included studies up to the year 2007, but the themes still seem to adequately express the focus of qualitative research. The body, which is otherwise taken for granted, has become an unreliable alien (Raaheim & Haaland, 2006), an overwhelming, inexplicable feeling of pain and fatigue disturbs the patients’ daily activities (Grape et al., 2017; Sallinen et al., 2011), and memory and concentration problems complicate performance of several daily tasks (Sallinen & Mengshoel, 2021). Thus, the patients are separated from the body they used to know and the life they used to live (Raaheim & Haaland, 2006). Life is described to be “governed by pain” (McMahon et al., 2012a) leading to worries and uncertainty about what is the right thing to do and what can be expected in the future (Taylor et al., 2016). Patients monitor their body carefully to find out what triggers the symptoms (Mengshoel & Grape, 2017), and adjustments may occur in terms of downregulation of daily life (Sim & Madden, 2008). As described, qualitative studies have focused on how FM symptoms are experienced and lived. However, given the long journey before arriving at the diagnosis, it seems reasonable to examine how patients experience the illness trajectory before ending up with a diagnosis of FM.

### Purpose

The intention was to learn in more detail how and when the illness started and how it evolved over time. Therefore, the purpose of this study is to explore the pre-diagnostic illness trajectory as narrated by individuals recently diagnosed with FM.

## Theoretical framework

Diseases are characterized by their known aetiology and pathogenesis, often accompanied by effective curative treatments (Blaxter, 2010; Bury, 2005; Cassell, 2004; Hofmann, 2008). In FM, there is no known cause, but there is extensive knowledge about various pathogenic mechanisms. However, its complexity does not lead to a definite treatment to normalize pathology, and some have named FM an “illness without disease” (Hadler & Greenhalgh, 2008). Illness describes a person’s suffering and its impact on lived life (Kleinman, 1988). As already illuminated, patients with FM experience medically inexplicable symptoms that disturb their daily life, but here, the purpose is to examine how a pre-diagnostic illness trajectory evolves and is given meaning in light of the present situation when having been recently diagnosed with FM. Another theoretical concept is sickness, which can be understood as “a social condition that applies to people who are reckoned by others to be ill or suffering from a disease. It refers to a particular status or role in society and is justified by reference either to the presence of disease or the experience of illness”(Radley, 2004^, p.^ ^3^). In society, the clinical invisibility in biomedical terms during the diagnostic process of FM creates uncertainty about whether a patient is sick or not, which may in turn affect a person’s credibility and self-esteem (Söderberg et al., 1999). Accordingly, the illness trajectory preceding FM can also be understood in terms of sickness. In accordance with Sontag (1990), sickness implies holding dual citizenship and having passports to both the kingdoms of ill and health. Thus, medical, personal as well as sociocultural aspects are likely to play a role in construing patients’ experiences of a pre-diagnostic illness trajectory.

Most qualitative interview studies about the illness experienced by patients with FM have applied thematic analysis, phenomenology, or grounded theory (McMahon et al., 2012b). These approaches are particularly well suited for examining patients’ experiences at the present moment while a narrative approach is well suited for exploring how a person construes a coherent meaning of their experiences by weaving together past and present experiences as well as future prospects during storytelling. Accordingly, people try to make sense of what happens to them by connecting past and present life experiences in order to create a coherent and meaningful story (Polkinhorne, 1995). In an anthology about narrative methods (Mattingly & Garro, 2000), the authors underline that storytelling about the past and the present cannot simply be seen as a reproduction of what has happened in “real life”; it is rather a reconstruction of what a person finds significant in the past for her/his present understanding. The narrator knows the present stance and is able to select relevant events or episodes in the past to clarify his/her points. Each individual speaks for themselves, but at the same time, the individual draws on the narrative scripts in our culture. The storytelling also invokes the voices of other people, and thus, characters are configured and play different roles within the story. The narrator balances between what he/she is willing to tell and what he/she thinks needs to be told. This does not mean that the narrator’s story is a strategic fiction, but it also has to be understood in light of the context in which it is told. A narrative analysis emphasizes the events and how they are used to clarify the narrator’s points to an audience (Riessman, 2008). By analysing what people tell, how they build up a story, which characters they include and the roles they give the characters, an overall meaning or a story’s plot can be reached (Squire et al., 2014).

## Methods

### Context, design and ethics

The present study is part of a project aimed at developing and trying out a new multidisciplinary patient education programme at the Hospital for Rheumatic Diseases, Lillehammer, Norway. This person-centred, recovery-oriented patient education programme aims to facilitate patients to explore and act upon their own experiences (Mengshoel et al., 2021). As an introduction to the programme, the patients are told that the programme is building on what is learnt from prior patients who have recovered from FM. On the first or second day of this programme, ten participants recently diagnosed with FM were interviewed. The HPs recruited 2–3 participants from the first groups of patients after asking for their willingness to be interviewed by the principal female investigator (the author). The participants were mostly referred to the programme by family physicians, and the FM diagnosis was confirmed or set by physicians at the hospital. The Norwegian Data Inspectorate for Research approved the study (no. 2018/57,956/3/EPA), and the participants signed a consent form after being informed orally and in writing about the purpose of the study, ensured anonymity, and their right to withdraw at any stage of the study without any consequences for their future treatment at the hospital.

### Interviews

The interviews were conducted in an office at the hospital. The female interviewer (author) presented herself as a health scientist at the University. Information about her professional background was excluded to avoid limiting the storytelling to what the participants might anticipate is relevant for a physiotherapist. After having provided information about age, education, family and employment status, an overarching question was asked: “Can you please take me back to when you think your illness started, and what happened afterwards?” The storytelling was sometimes rather fluent, with few disruptions by the interviewer except for prompting questions, such as: “Can you describe this situation further? What did you do next? What was it like for you then? What do you think about it now?” In several interviews, however, the participants hesitated and seemed not to know what to say, and they often responded with only a few sentences. In such cases, the interviewer rephrased what they had just said to facilitate further telling. This could also result in short descriptions that required the interviewer to repeat the rephrasing. Thus, the data material differs with respect to the interviewer’s role in co-authoring the participants’ stories. The interviews provided a rich, detailed data material referring to interviews lasting from 45 to about 90 minutes. The interviews were tape-recorded, and field notes were written to capture the immediate impressions of the interviewer.

### Analysis

The interviews were transcribed verbatim by the author. The recordings, transcripts and field notes constituted the data material for analysis. The initial general impression was that the interviewees told about a difficult life before the symptoms started, followed by years with recurrent episodes of symptoms that gradually unfolded to become more or less persistent. The storytelling seldom proceeded in a chronological order; often it jumped back and forth in time, as the interviewees kept returning to an episode they had recounted before to clarify their point further. Each narrative was examined in detail to identify and extract sequences about the life before symptoms, life with recurrent symptoms, and life with lasting symptoms. For each interview, the sequences were put in chronological order, and the temporal interrelationship between sequences about the same issue were analysed by examining whether a sequence was an introductory orientation, a description of the point, or an evaluation (Riessman, 2008). The sequences were coded, for example, as “strenuous life situations”, “bodily experiences”, and “hitting the wall”. Similarities and diversities across the analysed interviews were searched for (Riessman, 2008). Despite differing in detail and richness, the interviews were rather similar at an overarching level, and the analysis is therefore presented in three storylines based on the stories of the whole sample. When a quote consists of sequences from different parts of the interview, it is marked with […].

## Results

### Participants

Seven women and three men whose age varied from the early 20s to the early 50s were interviewed. Anne, the youngest one, lived alone with no children, while the oldest, Kirsty, was married, had adult children and a grandchild. The other participants aged 27 to 35 years (Tom, David, Christopher, Susan, Mary) and from 36 to 47 years (Joan, Lisa, Christine) lived with a partner and had children in nursery or primary school. Kirsty had a bachelor degree and a part-time office job. The other participants’ highest education was secondary school. The interviewees were employed part or full time in offices or shops (three had been promoted in the firm to become shop managers), owned a handicraft business, or did heavy manual work. At the time of the interviews, they were working either full or part time or were on temporary or long-time sick leave. Over the last two years, they had ended up with a diagnosis of FM.

### Strenuous life and alerted body prelude illness

The stories go back to life events in childhood, adolescence or early adulthood before the symptoms occur. They tell about demanding life events, such as death of a parent, high work load, physical abuse or psychological stress. The stories include parents portrayed as being in need of the interviewees’ care and comfort rather than as parents providing support. In this way, the interviewees portrait themselves as having been left to manage alone. For example, Susan goes back in time and tells about the time of her father’s death and what happened afterwards:

I lost my father to cancer when I was 16 years old. My mother could not cope with the loss of my dad and fell apart. She was given antidepressants and became unrecognisable. She actually became aggressive. I had to stop her using the pills. My mother didn’t get any financial support and couldn’t provide for us kids, and I started as a cleaner in a kindergarten after school. It is important in my family to work hard. Everybody is hard-working. So, I couldn’t finish secondary school, and simply, I have no education […]. I was extremely depressed by the loss of my dad, and my atopic eczema flared up when he died. Yes, I had sausage fingers and couldn’t use my fingers. They swelled up and were huge. I’ve heard that grief can exacerbate disease. I’m thinking this triggered my disease. I’ve started to think about it now when I’m not at work.

Susan tells about a sudden change in her life that forces her to take over the responsibility for her family. She starts to work hard, and apparently this is more or less taken for granted by her family. Moreover, she clarifies her point that psyche and soma are interconnected. The interviewees’ stories also include long stressful life events. For example, David tells about his parents’ divorce and his choice as a 12-year-old boy to live with his alcoholic father to avoid the strict rules and restrictions of his mother:

I kind of have an anchor hanging behind me – a chain that I can’t completely get rid of. I’ve been worried a lot. My dad had diabetes, and I spoke with the emergency care once a week, right. He’d been drinking and taken a double dose (insulin), and then quick action was needed. I was so worried. I was up at night checking if he was alive, quite simply. What I feared finally happened. I found him in a coma, and after a week on a ventilator he died […]. So, my body has been on alert for many years. It doesn’t have to be so now, but it still is. Old habits are difficult to reverse, and things happening physically in the body also become a habit […]. Yes, I chose to live with dad, stupid that I was, right. I could eat crisps and watch movies whenever I wanted. If I wanted time off from school, he wrote a message in the book. I don’t think there are a lot of children who would say no to such an opportunity. But I can’t blame my parents or myself.

David tells that due to long-time worries for his father, a bodily alertness develops that is impossible for him to reverse. For David and the other interviewees, intolerance to light, noise, physical and mental stress is common today. The interviews also tell about an innate alerted body exemplified by “always” having difficulties in “calming down” and a need to withdraw to peaceful places. Kirsty recounts that she was always a sensitive child and verifies it by referring to others’ observations of her increasing pallor:

My parents told me that they couldn’t send me to nursery school. We lived in a small village at that time, and both my mother and father worked. It wasn’t like this with the other housewives, they stayed at home. But my parents had to put me in nursery school. However, it was so noisy there, and I became so tired. So they found a nanny. Yes, I had her as long as we lived there […]. I’ve never liked to go to cities or go out to buy clothes. Then, I’m just grey in the face, and I was already like this as a child. Even before the pain started. I was very sensitive to sound, and I’ve always slept lightly. It doesn’t take much to wake me up.

This storyline draws on a cultural narrative that a strenuous life marks the body and a medical narrative of an innate bodily vulnerability. The central character in the storytelling is the narrator, portrayed as a responsible, persevering individual. This picture is strengthened by portraying parents in need of their care and support. One may read this story as parents and other adults failing them, but they either do not say that or underline that the parents cannot be blamed.

### Recurrent pains unfolding to become lasting and complex illness

When describing the time before the illness, several episodes of recurrent pain are gradually woven into the stories. The occurrence of pain is not described as definite events, but is displayed in general terms. In this part of the storytelling, physicians enter the scene. They are often portrayed as not listening to the interviewees, ignoring their pain or not knowing what to do to help them, as exemplified by Kirsty:

I’ve had a lot of joint problems, starting with knee problems in my early teens, a lot of back pain that nobody understood. I’ve kind of always heard that there are no kids having back pain, so you don’t either, no! So that’s how the years have gone by. So, I have been really tired during these years, and I had a lot of inflammation too. Yes, I still have […]. It escalated, and the doctors said that I had a reactivation of kissing disease in adulthood. Something I didn’t know I’d had. But after that it got worse, more infections. Very unstable immune system, I would say.

Kirsty ascribes her pain episodes to an impaired immune system, and it is mentioned that doctors also ascribe pain to biological changes, for example, by telling that pain is “only growing pains” or relates to overload from heavy work. The interviewees are told that the pain will end when they stop growing or if they take it easy for some time. But they do not recover fully. Lisa explains:

I’ve been in pain more or less for 15 years, and it started with painful wrists and ankles. I was seeing the doctor all the time, and she said it was inflammation. There was a lot of physical work and long days at job. I used to work in a kitchen. It was too much for me, and I had two small kids at home as well […]. I didn’t want sick leave, and the doctor told me it would pass and I would become well again. I believed the doctor. Maybe it’ll be over in a week or a month? But it was not.

The stories continue by describing how the interviewees push themselves to endure recurrent pain moving from one location to another. Gradually, the pain appears more frequently, lasts longer and at last becomes more or less persistent, spreading to the whole body. Along the way, several other discomforts appear, especially an unusual feeling of tiredness. The interviewees explain that this occurs in parallel with multiple, accumulating stressful life events, such as surgery, difficult childbirths, non-sleeping babies, seriously ill or disabled children or family conflicts, which they think contribute to escalating their ill-being. For example, Mary goes through an unplanned dramatic caesarean section; shortly afterwards her mother gets a cancer relapse, her baby keeps her awake at nights, then her grandma dies, and her husband is involved in a car accident and loses his driving licence, resulting in her having to take over the daily nursery school run. Mary tells what this led to:

I thought I’d had a flu. Because it felt that way. It was the worst that I’d ever had. So, I stood in the shower on three consecutive nights because I was in such terrible pain. I stayed at home for a couple of days, but I didn’t get a cold or fever or anything. So, I went back to work. […]. I really swelled up in my hands and especially in my feet, plus my toes were very red. So I’m sure there was inflammation and stuff like that. So, I was referred to a rheumatologist. Blood tests were checked and so on. But the doctor said you’re perfectly healthy. You don’t have rheumatoid arthritis or anything like that. But there was something wrong!

At this stage, the stories also include accounts of difficulty calming down and falling asleep, and if the participants think they sleep well, they still wake up unrefreshed in the morning. They feel exhausted. They reach a point where illness disturbs their everyday life to such an extent that they no longer manage to ignore it or fulfil their daily obligations. Nevertheless, health professionals tell them to stay active, and they do their best to follow the advice. At last, they either realize that this cannot go on any longer or arrive at a point which they describe as “hitting the wall”. Susan explains:

I noticed it a year before it stopped. I was always tired and in constant pain. I was hurting from my head to under my heels. I did not have any energy in my body anymore. I became slower and slower, yes! Continued to work. I endured a lot and thought it was going to get better, but it didn’t. Spent all my energy at work to get the income we needed […]. After a haemorrhoid operation, I got painkillers that enabled me to relax. When I quit the pills, it was just like I went straight down. I just collapsed completely. I just slept all the time, slept 24/7. I went to the doctor because I didn’t think it was right, but he did not understand. It was terrifying.

This storyline displays an escalating illness trajectory portraying a body gradually becoming more and more painful, exhausted and non-obedient. They are despairing, and it more or less ends with a full stop. Throughout this storyline, the participants continue to portray themselves as responsible, persevering persons adhering to experts’ advices, but they are at last forced to give up by their illness. At the same time, their portrayals of the doctors position the interviewees as wounded by misguidance, and thus, they continue to be failed by others. This storyline is very much in line with a common narrative circulating among patients with medically unexplained symptoms.

### Diagnosed but still uncertain presence and future

The stories continue after the event of “hitting the wall” by describing a slight improvement. However, the body remains painful, stiff, exhausted, and intolerant to stimuli, and it continues to actively resist what they want to do. They do not know what they can tolerate anymore as one day, they are able to do a lot, whereas on another day, they cannot do anything. Also, they find it paradoxical that they feel well as long as they stay active, but afterwards it is “payback time”. This does not make any sense to them.

Finally, either the family physicians decide, or the participants urge the physicians to refer them to medical specialists for diagnostic evaluation, and they are diagnosed with FM. The participants have close family members who have either rheumatic inflammatory diseases, or more often, parents, grandparents or siblings with FM. Despite this, they are surprised when diagnosed with FM. They explain that they do not know much about FM, but have considered FM a “nonsense diagnosis”, “laziness label”, or a “sack for everything and nothing”. Moreover, they have believed that the FM diagnosis was reserved for “elderly worn-out women and not for young people”, but at the patient education programme they have just attended, they are together with other young people “who are in the same boat”. This makes them rethink the meaning of FM, as Tom explains:

It was a relief to get a diagnosis and a confirmation of the years I’ve had this, you could say. I don’t know anyone who has FM, and I’m talking to these people (peers in the programme). I recognise myself. Some have had it for a short time, and others much longer than me. Some are disabled, and others are not. Very different! I find that even though I recognise myself in a lot of what they say, FM has several different implications for people. On this basis, I think I’m going to learn a lot by talking with those in the same boat as me and the qualified professionals here. Get tips and advice about what I can do.

Like Tom, most interviewees accept the diagnosis, and they are satisfied with being diagnosed at last. They had expected that a diagnosis is accompanied by effective treatment, but this hope evaporates when they realize that they have already found the usual treatments offered for FM to be of little help. However, their doctors refer them to a patient education programme designed particularly for people with FM. They hope this will enable them to see their illness with “new glasses” and find solutions for how to improve their situation. In this part of the story, they say they have confidence in their present doctors who are portrayed as supportive because they believe in them, refer them to specialists and treatments, and write sick-notes without any questions. In this storyline, the coaches at the social security office are portrayed as their opponents. They explain that due to the invisibility of their illness, they continue to push them to stay on at work. On behalf of herself and her peers, Christine expresses this opinion:

We would have it easier if it was visible. Like my coach at the social security office said, ‘I don’t see you’re sick, and I don’t think you’re sick!’ But those who have cancer then, if they’re not losing their hair, not everyone will see that they have cancer either, right. I said (to the coach) it’s terribly dangerous to put yourself in this position and overrule my doctor’s judgment that I am ill.

The interviewees say that some future recovery might be possible, but before thinking about the future, they first have to find solutions to manage the present situation. They express a fragile hope that new insights will enable them to find ways to manage their illness at present. In line with others, David’s account reveals the opinion that they have quite enough to do to manage one day at a time, and they are unable to have any expectations for the future:

I don’t know what the future holds. No one does! In the past, I had a lot of expectations for the future, but I don’t anymore. I don’t plan much anymore. But I hope I will still be at work and will have found a way to live with less pain. At least that’s my vision. As regards small details, that has to come later. But if I find ways to have less pain, then I will continue to manage regular things.

This storyline expresses an ambivalence. On the one hand, it expresses satisfaction with finally arriving at a diagnosis, being referred to a FM programme, and a hope of learning from other patients and qualified health professionals. On the other hand, the storyline includes uncertainty about how to manage the present illness situation and what the future may hold. Thereby, the storyline lacks a solution or an ending. Here, the interviewees portray themselves as uncertain and vulnerable, but they also express fortitude and willingness to continue their endeavours to make the situation better for themselves. Thus, their portrayal as responsible and persevering individuals is carried forward. However, since a narrative about effective treatment related to the diagnosis of FM is still lacking, they are simply left to hope to figure it out by learning from others.

## Discussion

The present study’s narrative plot is “a long, winding trajectory of suffering without any definite start or ending” expressed through the following three storylines: “Strenuous life situations and alerted body preluding illness”, “Recurrent pains gradually unfolding to become a lasting, complex illness”, and “Diagnosed but still an uncertain presence and future”.

British patients considered that FM is caused by physical assaults, emotional trauma and distress, as well as stress and vulnerability (Furness et al., 2018). Such probable causal events are incorporated in the present interviews as well. The British study, however, was not designed to capture how the perceived causes were embedded within the respondents’ illness trajectory. Thus, the present study adds to the prior findings, suggesting that the participants do not depict one distinctive cause of FM; rather, they portray a long process of recurrent strenuous personal and social events as forerunners of illness as well as continued escalation of recurrent episodes of pain into FM. The present narrative does not point at any definite event starting the illness. Instead, the interviewees seem to draw on existing narratives about strenuous life triggering diseases and a sensitive body making people more vulnerable to diseases. The present narrative also raises the question about how to decide when FM actually starts. For example, does FM start with the experiences of strenuous life situations early in life, with the first illness episode, when the repeated illness episodes become more frequent, long-lasting or persistent, at the time of ’hitting the wall’, or when diagnosed with FM? Actually, the present narrative suggests that FM is the endpoint of a long trajectory of suffering, indicating that early interventions might have prevented the development of FM.

Mapping symptoms is often incorporated in diagnostic assessment in order to provide hints for further medical assessments (Eriksen & Risør, 2014) or for the purpose of deciding what to focus on in treatment (Ahlsen et al., 2018). Years ago, Malterud (2000) argued that doctors should appreciate patients’ experiences as a source of knowledge helping patients and doctors to concretize the situation and collaborate in finding appropriate solutions. This seems relevant as life experiences in the form of challenging life situations, previous illness, and negative experiences with helpers and treatments add to a person’s suffering (Cassell, 2004). At present, the narrative plot “a long, winding trajectory of suffering” captures such elements. As shown here, understanding a person’s suffering and illness cannot be separated from a person’s life story by solely mapping symptoms. Neither can a narrative be separated from who is the audience. Portrayal of themselves as persons with stamina to withstand the troubles they have met in life affects listeners, convincing them that these are ill persons worthy of help at last. Nevertheless, the life events cannot be seen as fiction; they refer to events that occurred in their lives which they today find significant in explaining their present situation to an interviewer having a background as health professional.

It can be difficult to understand patients’ narratives as they may seem chaotic, broken or incoherent. Bülow (2008) argues that such narratives can signify that the storyteller has not yet fully developed a meaning of their experiences. The early reading of the present interview material gave the impression that the narratives were incoherent and did not necessarily express a clear meaning. However, when sequences addressing similar issues throughout the narratives were organized together, it was gradually revealed how the sequences were interconnected. This detailed analysis led to a gradual understanding of the narratives’ overall meaning of suffering that extended the concept of illness, as it also included telling about their suffering ahead of the illness. In addition, some participants more or less finalized their story by summing up what they had told, suggesting that they had created a meaning of their experiences during their storytelling. After the interview, some said, unsolicited, that they had never been asked to tell their whole story before, and telling it now had made them rethink why they had become ill. As noted by Kleinman (1988), developing such a meaning is crucial for a patient in order to come to terms with illness and manage to live with it.

Prior studies have shown that patients may have several negative experiences from consulting health professionals, such as not being listened to and taken seriously (Mengshoel et al., 2018). This is also the experience of the present participants. They say they were pushed to do what they think contributed to making their illness worse. Not complying with the recommendations of medical and social authorities can be risky for patients as they may lose help. Doctors and coaches at the social security office were portrayed as prominent characters in the present participants’ narratives. They could be described as both opponents and supporters. The antagonists are described as counteracting persons who ignored the participants’ experiences and told them to do things which they found impossible or meaningless. In such cases, the meetings with helpers were also seen as causal events contributing to more suffering.

According to Cassell (2004), hope for the future is necessary for living a successful life, and without any hope, suffering becomes worse. At present, the hope is for a better present rather than future, indicating that they are still struggling to remake a tolerable life. Indeed, effect studies give little promise of curing FM or of considerable, lasting symptom relief (Macfarlane et al., 2017). On the other hand, some have recovered from FM (Grape et al., 2015; Mengshoel & Heggen, 2004; Sallinen et al., 2012; Wentz et al., 2012). The existing pessimistic narrative about FM can thus be seen as somewhat more optimistic. As described by Mattingly (2010, ^p.^ ^5^), hope is “involving the practice of creating, or trying to create, a life worth living even in the midst of suffering, even with no happy ending in sight”.

### Methodological considerations

A methodological strength in the present study is the detailed, rich data material about the illness trajectory before ending up with FM, and thereby the study provides strong information power (Malterud et al., 2016). Despite great detail and variability, a shared narrative about the long, winding trajectory of suffering without a definite start and an uncertain ending made sense of the whole data material. The trustworthiness of the findings depends largely on the researcher’s efforts in letting the participants tell their version of the illness trajectory, and the researcher’s ability to raise critical questions on her own role and interpretations in each step of the research process. The interview situation could be challenging as particularly at the beginning of the interviews, several of the interviewees had only little to say. However, it seemed fruitful to rephrase what they said instead of asking questions. It was anticipated that they would have a lot to say about symptoms; instead, they told about how life was disturbed or corrupted. During the analysis, theories of illness and narratives were actively used to raise critical questions for the interpretations. Hopefully, the paper provides enough detailed information to clarify the coherence between how the interviews were conducted, what was told, and the interpretations.

With respect to transferability, the question is whether the pre-diagnostic illness trajectory experienced by relatively young individuals with low education referred to a specialized hospital can be transferred to, for example, older or more educated patients or to patients in other clinical settings within health services. It seems reasonable that a long-lasting pre-diagnostic illness trajectory may occur independent of age, health care settings and countries, as shown in a multinational survey (Choy et al., 2010). However, it seems plausible that there can be other pre-diagnostic illness trajectories as well, but this is a question for future research. A narrative study will never claim that it captures all circulating narratives about a phenomenon.

### Clinical implications

The present study underlines the importance of clinicians listening to and learning from a patient’s life story. Madden and Sim (2006) argue that telling narratives are important for a patient to create a meaning of experiences, accept and manage changes and reorganize life to adjust to illness. It is also argued that the meaning of suffering is determining how people act (Cassell, 2013). Thus, storytelling is therapeutic (Charon, 2006; Clark & Mishler, 1992; Kleinman, 1988). It also seems plausible that the present narrative can help clinicians to recognize and make sense of what patients tell, as well identify the need of treatment at an earlier stage in the illness course to prevent the development of FM.

## Conclusions

The three storylines portray a long, winding trajectory of suffering starting before the debut of illness, and an illness gradually unfolding to become persistent and overwhelming. Finally, a diagnosis of FM is arrived at, but what the person’s situation will be is uncertain. This study indicate that the understanding of illness cannot be separated from the person and her/his lived life.

## Data Availability

Audiotapes and transcribed interviews are stored on Secured Data Server for Research at the University of Oslo, Norway, and to protect individual privacy, the data material cannot be shared publicly.
